# Comparison of outcome of TIP urethroplasty with or without Buck’s Fascia repair

**DOI:** 10.1186/s12894-024-01468-x

**Published:** 2024-06-27

**Authors:** Raashid Hamid, Aejaz A. Baba

**Affiliations:** Department of Paediatric Surgery/Urology and Neonatal Surgery, Sher-I-Kashmir Institute of Medical Science, Old Library Room No 2, Srinagar, 190011 Jammu and Kashmir India

**Keywords:** Bucks Fascia, Snod grass urethroplasty, Meatus, Uretherocutaneous fistula

## Abstract

**Objective:**

TIP is the most common preformed type of Urethroplasty. The intermediate barrier is used as a waterproofing layer to prevent fistula formation. Many tissues have been utilized as a barrier layer, with varying success rates. The search for a better intermediate layer will continue. In this study, we aim to evaluate the role of Buck’s Fascia as a covering for the neo-urethra to prevent fistula formation in patients who underwent Snodgrass Urethroplasty.

**Methods:**

This prospective study was conducted between 2018 and 2022. Patients were randomly assigned to either Group ‘**A’** or Group ‘**B’**. Group A included patients who underwent the Snodgrass procedure with a Buck’s Fascia cover, while Group B included patients whose neo-urethra was covered with the dartos flap. These patients were closely monitored for the development of short- and long-term complications in both groups, and the results were recorded.

**Results:**

The study involved 164 patients, who underwent midpenile and distal hypospadias repair using the Snodgrass technique. In Group ‘**A’** (84 patients), the neo-urethra was covered with Buck’s Fascia, while in Group ‘**B’** (80 patients), the neo-urethra was covered with the dartos flap. The mean age of the children was (23.06 ± 16.12) months in group ‘**A’** & (26.06 ± 14.07) months in group ‘**B’**. mean operating time was (40 ± 11.43) minutes, in Group ‘**A**’, and (70 ± 17.43) minutes, in Group ‘**B’**. Meatal stenosis occurred in 3.57% of children in Group ‘**A’** and 10% of patients in Group ‘**B’**. Urethral fistulas were encountered in 2.35% of cases in Group ‘**A’**and 10% in Group ‘**B’**. The difference between the groups was statistically significant.

## Background

Surgical procedures for hypospadias are both numerous and intricate. Among these, the TIP urethroplasty stands out as the most commonly utilized method for distal hypospadias repair. Controversy has persisted regarding the relative success and complication rates of these procedures [1]. Achieving good cosmesis, with a meatus resembling a slit, has always been a shared goal for both surgeons and parents. The primary objective of urethroplasty is to reduce the incidence of fistula formation and meatal stenosis [2, 3]. Various intermediate layers, including spongiosum, dartos, and T.V.F, have been employed to mitigate these complications. In the existing literature, different authors have detailed the advantages and drawbacks of these intermediate barriers, yet none have been hailed as ideal. Moreover, fibrin glue has been studied and compared with the dartos flap [3]. There is still debate over which material provide the most effective barrier to prevent UCF. Since 2016, efforts have been made to utilize Buck’s fascia and the glans as integral covering tissues, forming an intermediary layer to envelop the neo-urethra in TIP. Varied outcomes have been reported across different centers, with an overall consensus indicating superior efficacy compared to using simple pedicled dartos fascia [4]. In a retrospective analysis by Zhou Qian et al., concluded that the utilization of Buck’s fascia to cover the neourethra effectively decreases the occurrence of urethral fistulas in TIP surgery [5]. They also emphasized the importance of considering Buck’s fascia quality and suture tension to prevent the development of urethral strictures. In our study, we aim to demonstrate and compare the use of Buck’s Fascia as an intermediate barrier between the neourethra and the skin. Our focus will be on comparing Buck’s Fascia repair with the dartos flap repair.

## Methods

This study was conducted in the Department of Pediatric Surgery at SKIMS between September 2018 and September 2022. The sample size was selected to allow for meaningful evaluation of the intervention. Ethical approval for the study was obtained from the institutional ethical committee (IEC/SKIM/23/2/2021), and informed consent was acquired from the parents. Patients with distal hypospadias were categorized into two groups. Group ‘**A’** comprised patients undergoing Buck’s Fascia repair, while Group ‘**B’** included patients with the dartos flap as an intermediate layer. The variables measured included complete Cleft, incomplete Cleft, shallow, width of the Urethral plate, presence of chordee, absence of chordee, superficial chordee. Two surgeons performed the procedures for each group. We used random-numbers table that generated the random sequence. All the children underwent a comprehensive physical examination, which encompassed an evaluation of phallus length, chordee, urethral plate, glans size, and skin condition. Inclusion criteria were distal hypospadias, age less than 12 years. Exclusion criteria were previously operated cases, severe chordee, UCF, circumcised children proximal hypospadias. Baseline investigations were conducted for all patients.

## Operative techniques

A circumferential sub coronal incision was made proximal to the ectopic meatus. The penis was degloved, and chordee correction was performed, with an additional longitudinal incision made in the upper part to create a wider upper section. The neourethra was constructed using a 6 F/8F silicon catheter and 6 − 0 PDS or Polydioxanone sutures, with continuous sutures used for tubularization. A dartos flap, complete with its blood supply, was harvested from the prepuce and transferred from the dorsum to the ventrum through a hole in the pedicle, effectively covering the neourethra. At the conclusion of the procedure, the glans wings were approximated without creating tension in two layers, and the skin cover was completed. A simple gauze dressing was applied and left in place for 3–4 days. The antiseptic dressing was removed on the 4th postoperative day. Patients were discharged on the 5th to 6th postoperative day with the catheter in situ, and they were advised to follow up weekly. The catheter was removed on the 8th to 10th postoperative day.

In Group **A**, Buck’s Fascia was used as an intermediate layer. After degloving the phallus and making a midline incision in the UP (urethral plate), the UP was tubularized over the stent using 6 − 0 Vicryl/PDS with continuous sutures. Following the repair of the first layer, proximal to the corpora spongiosum disjunction, the Buck’s Fascia overlying the spongiosum was identified and traced as a “V”-shaped defect, with each limb of the “V” seen laterally to the spongiosum, representing the free medical margin of the deficient Buck’s Fascia. This Fascia was sutured in the midline without suturing the spongiosum, starting just proximal to the first suture line. The suture line was continued distally into the glans and then, without knotting, carried backward to suture the glanular skin. The skin was closed using 6 − 0 Vicryl with interrupted horizontal mattress sutures, and dressing was applied. The operative time was measured upon procedure completion.

Both groups were monitored for early and late postoperative complications, including edema, hematoma, wound infection, glanular dehiscence, and early fistula formation. During follow-up after catheter removal, the following complications were noted, including urethrocutaneous fistulas (UCF), meatal stenosis, and residual chordee.

### Group ‘A’

**Fig. 1:**
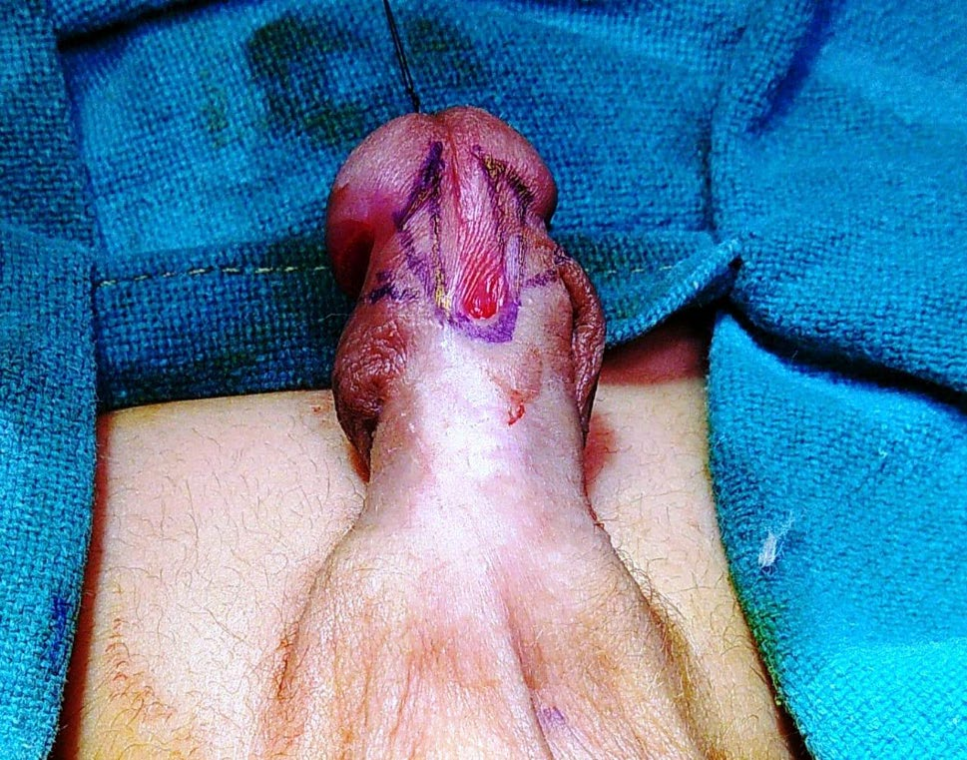
Making of incision around the ectopic meatus

**Fig. 2:**
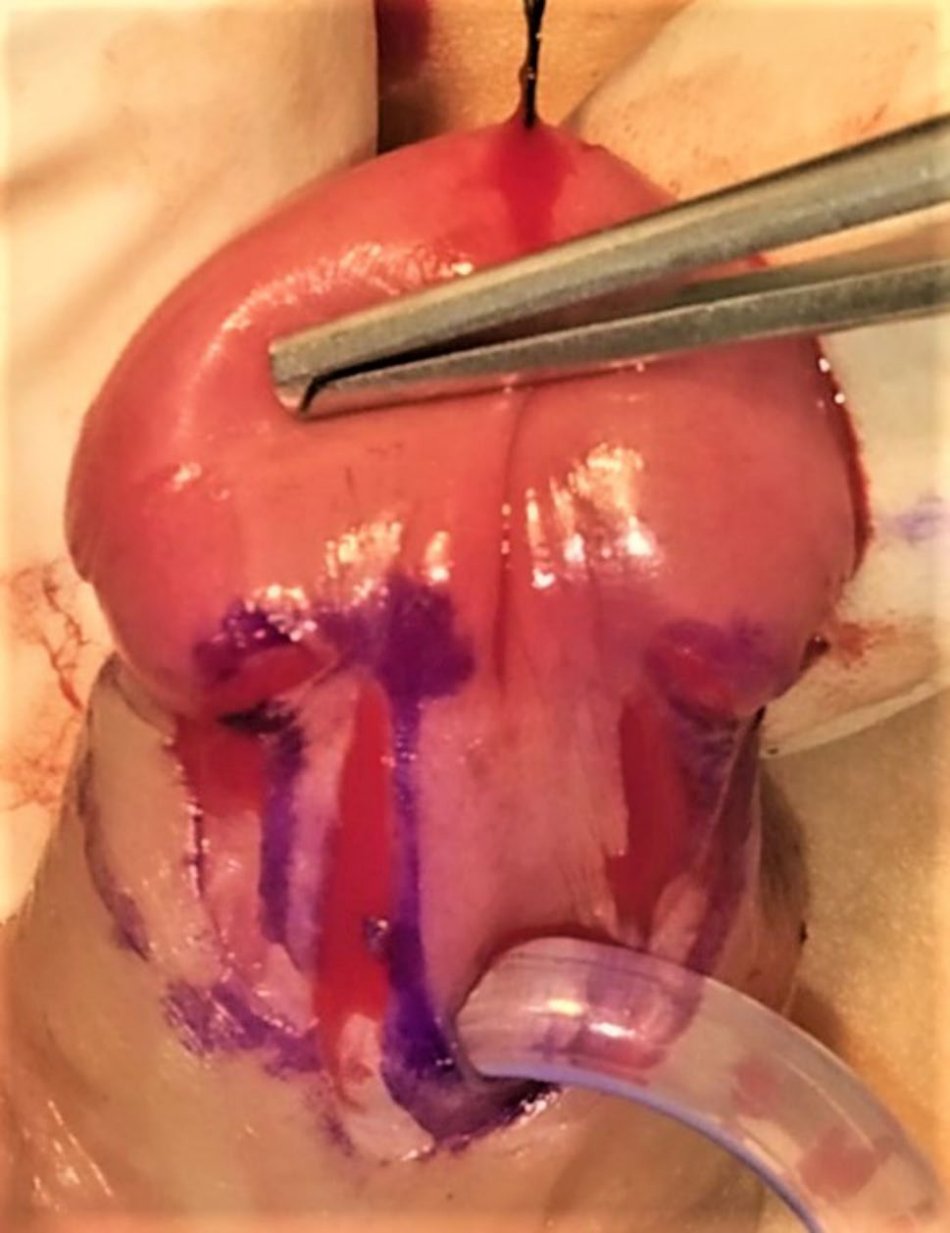
Incision made along the marked line and circumferentially in sub coronal area

**Fig. 3:**
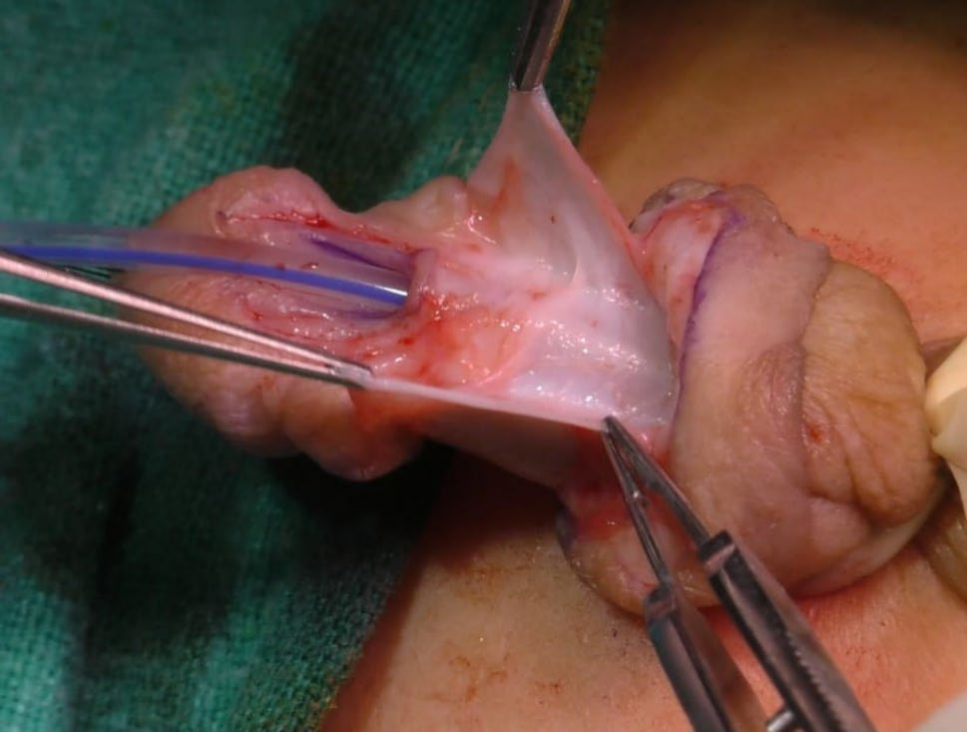
Demonstration of Bucks fascia

**Fig. 4:**
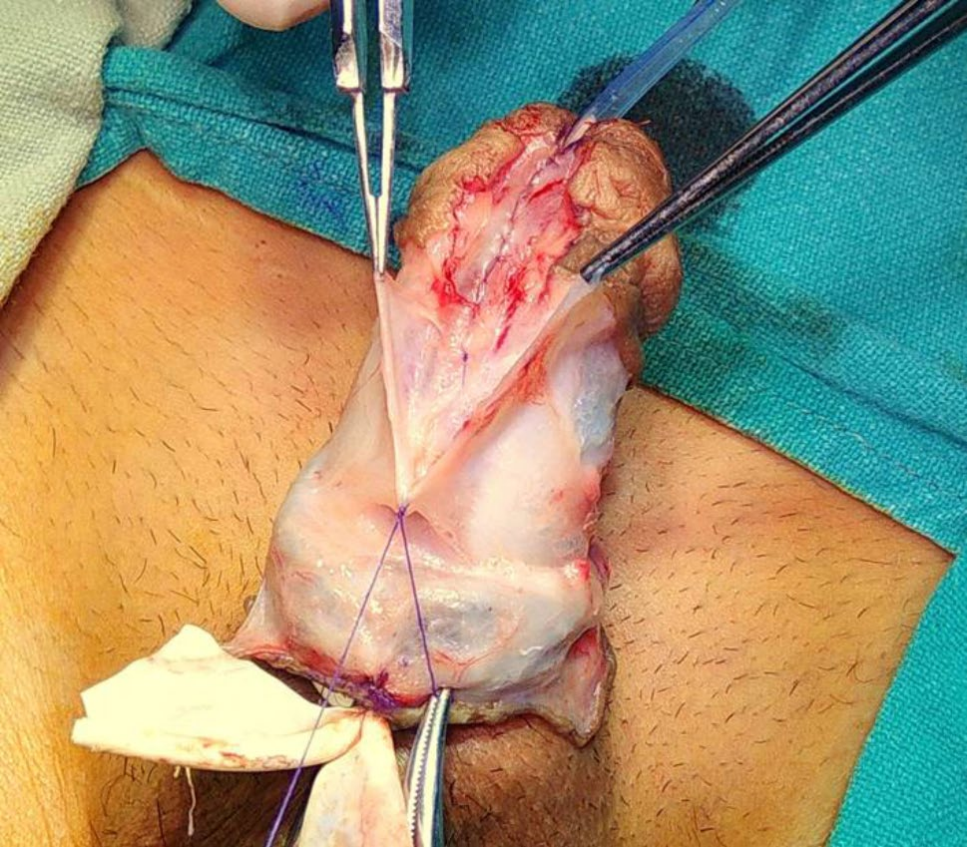
Closing Bucks fascia over the neo Ureththra

**Fig. 5:**
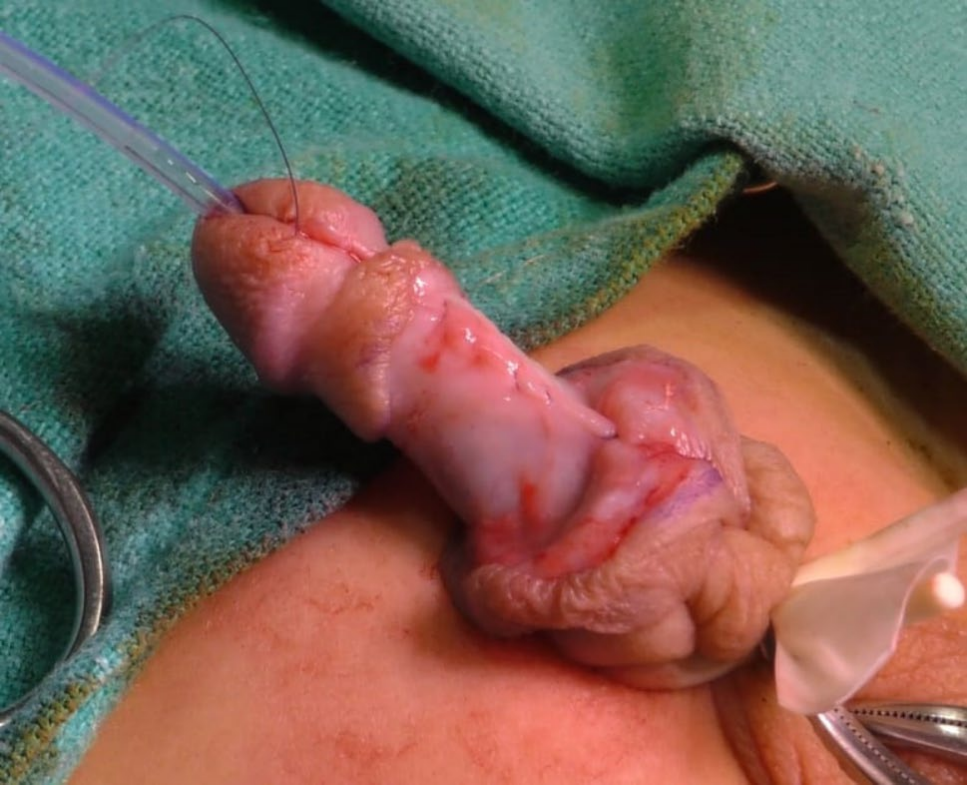
Complete covering and Glanoplasty

This group comprised 94 patients in whom Buck’s Fascia repair was performed (Figs. 1, 2, 3, 4 and 5).

### Group ‘B’

This group included 80 patients in whom the dartos fascia was used as an intermediate layer.

The operated case after discharge from the hospital where followed weekly for 1month, monthly for next 3 months and then 6 monthly thereafter.

### Statistical analysis

All data were collected, tabulated, and statistically analyzed using SPSS for Windows. Qualitative data were expressed as n (%) and analyzed using the chi-square test and 2 × 2 tables. A significance level of *p* < 0.05 was used to determine statistical significance. For qualitative data, the standard deviation was calculated, and data were compared using Student’s t-test.

## Results

The mean age of the children was (23.06 ± 16.12) months in group ‘**A’**&The mean age of the children was (26.06 ± 14.07) months in group ‘**B’**. The location of the ectopic meatus was found to be coronal, sub coronal, and distal glanular in 34, 33, and 17 patients in Group ‘**A’** and 32, 32, and 16 patients in Group ‘**B’**. Patients in both the groups had distal hypospadias mild to moderate chordee. The mean width of the urethral plate in group A & B was 6.88 ± 1.40 mm & 6.78 ± 1.45 mm respectively. The mean operative time was (40 ± 11.43) minutes in Group ‘**A’**, and (70 ± 17.43) in Group ‘**B’**. The follow-up duration ranged from 2 to 21 months in Group ‘**A**’ and from 1 to 24 months in Group ‘**B’**. In Group **A**, significantly fewer cases of glanular edema or discoloration were observed compared to Group ‘**B**’. The incidence of hematoma and wound infection was lower in patients who underwent Buck’s fascia repair (*p* < 0.05). The median follow up time was 18months in Group ‘**A’**& 20 months in group ‘**B’**. In Group ‘**A**’, one patient developed meatal stenosis, whereas five patients developed meatal stenosis in Group ‘**B**’ (p < 0.861). Urethrocutaneous fistulas (UCF) occurred in only two patients when Buck’s fascia was used as a barrier layer, while eight patients in Group ‘**B**’ developed UCF. Three cases of meatal stenosis, which responded to dilatation, were observed in patients who underwent meatoplasty for UCF repair (Tables 1–4).

**Table 1:** Table showing comparison of patient characteristics

Location of Meatus	Group A n( %)	Group B n( %)	P value
Coronal	34(40.47)	32(40)	0.334
Subcoronal	33(33.28)	32(40)	0.421
Distal	17(20.23)	16(20)	0.342
Total	84(100)	80(100)	

**Table 2:** Table showing local characteristics in two groups

Features	Column1	Column2	Column3
Degree of Chordee/Cleft	Group A n(%)	Group B n(%)	P value
No Chordee	32(38)	29(36.25)	0.897
Superficial Chordee	22(26.18)	21(26.25)	0.686
Deep Chordee	12(14.28)	14(17.5)	0.825
Complete cleft	6(7.14)	6(7.50)	0.134
incomplete cleft	9(10.71)	8(10.00)	0.432
Shallow UP	3(3.57)	2(2.50)	0.082
Total	84(100)	80(100)	

**Table 3:** Table showing early postoperative problems in two groups

Early Post operative problems	Group A n(%)	Group B n(%)	P value
Oedema	6(7.14)	15(18.75)	0.002
Glans discoloration	5(5.95)	10(12.5)	0.042
Glanular dehisence	1(1.19)	5(6.25)	0.001
Hematoma	3(3.59)	8(10)	0.942
Wound Infection	3(3.59)	5(6.25)	0.913

**Table 4:** Table showing comparison of complications in two groups

Column1	Column2	Column3	Column4
Follow-up Complications	Group A n(%)	Group B n(%)	P-value
Urethrocutaneous Fistula	2(2.35)	6(8)	0.0354
Meatal Stenosis	3(3.57)	8(10)	0.042
Persistent Chordee	1(1.19)	2(2.50)	0.0057
Penile Torsion	2(2.38)	3(3.75)	0.0098
Total			

## Discussion

The introduction of an intermediate layer in urethroplasty has significantly reduced the formation of urethrocutaneous fistulas (UCF). Durhan Smith was the first to introduce an interposition layer between the neourethra and the cutaneous sutures. Various types of waterproofing layers have been explored over the years, including Snow et al., tunica vaginalis wrap, Reticketal et al., dorsal prepuce flap, Motiwala dartos flap, and Yamakatasupermatic fascia flap [6–9]. These authors emphasized that the primary change in technique was the use of a waterproof barrier layer. The quest to reduce complications post-urethroplasty, especially UCF, persists. Thus, we compared the dorsal subcutaneous flap with a novel approach: Buck’s fascia closure over the neourethra.

Some scholars have proposed the concept of using Buck’s fascia coverage to restore the normal anatomy of the penis. This material possesses a tough texture and provides clear coverage, with the capability to significantly reduce urethral tension and the incidence of urethral fistula [4, 10]. It also maintains anatomical integrity from the two flanks of the head of the penis as a whole, preserving tissue continuity at the coronal sulcus and contributing to the inhibition of coronal fistula occurrence [5]. Buck’s fascia (the deep fascia of the penis) anteriorly splits to cover the corpus spongiosum. It can be easily identified over the spongiosum, traced distally, and brought to the midline over the neourethra. We used this layer to cover the neourethra without suturing the corpus spongiosum, as suturing the corpus spongiosum could potentially interfere with vascularity. This fascia is readily available in all cases and does not require repeat dissection. It provides a smooth covering for the neourethra.

As demonstrated in our series, the Buck’s fascia repair is significantly less time-consuming as it requires minimal dissection. The operative time was significantly lower in group ‘**A’**, similar to the results by Zhou et al. &Albilyosar A et al. [5, 11]. Early post-operative issues such as glanular edema, hematoma, and infection were significantly less frequent than with the dartos flap barrier. This observation may be attributed to the shorter operative time, minimal dissection of glanular wings, and the absence of the need to separate the dartos flap from the skin. As described in our procedure, the spongiosum remains undisturbed while waterproofing with Buck’s fascia, which may further reduce the chances of edema and hematoma.

Given that the primary objective of urethroplasty is to decrease the occurrence of UCF, many authors are actively searching for an ideal interposition barrier. The incidence of UCF was significantly lower with Buck’s fascia than with the dartos flap procedure. In a multicenter Chinese study by Yin Zhang et al., the incidence of complications included fistulas (5.2%), dehiscence (0.6%), strictures (1.6%), and diverticula in (0.7%) of cases [4]. The rate of complications was consistent with the results of our study. In a study conducted by Zhou Qian et al., compared the surgical outcomes of Buck’s fascia and Dartos fascia, revealing a lower incidence of urethral fistula (9.4%) with Buck’s fascia coverage compared to Dartos fascia coverage (29.8%). The odds of urethral fistula were reduced by 5.1-fold (95% CI 1.09–25, *P* < 0.05) with the use of Buck’s fascia [5]. The results of our study was almost same in terms of reduction in the rate of fistula formation. Snow Cartwright described the use of TVF as a waterproof layer over the neourethra [12]. The dorsal subcutaneous flap by Retiket and TVF (TVF) has been described as a vascular flap with simpler techniques and lower complication rates in urethroplasty [7]. As observed from one study, application of Buck’s fascia had a low UCF formation rate compared to the dartos flap and all other interposition layers described in the literature. The incidence of UCF in our series was 2.8%, well below the accepted standard of approximately 5% for anterior hypospadias, as reported by many authors like Keays MA et al. ,AmilBhatet al. &Bhat A, Mandal AK et al. [13–15], who used Bucks fascia as the intermediate barrier in urethroplasty. A Chinese multicenter study revealed a 4.9% incidence of urethral fistula with the application of Buck’s fascia coverage in TIPU, strongly validating the effectiveness of this method. The incidence of urethral fistula (9.4%) with Buck’s fascia coverage in a study by Zhou Qian et al. Comparing the result of our study the rate of fistula formation was slightly less than that observed byZhang, Y&Zhou Qian et al.

Meatal stenosis was higher in group ‘B’ (2.35% of cases in Group ‘**A’**and 10% in Group‘**B’**). We hypothesize that this observation could be due to the need for slightly more mobilization of the glanular wings to accommodate the dartos fascia. The significantly lower rate of meatal stenosis in Buck’s fascia repair could be attributed to glanuloplasty without extensive mobilization of the glanular wings and less interference with blood supply. Previously, glanular wingless procedures have been used in GAP procedures with excellent results [16]. Meatal stenosis was reported in 1.6% and 3% by authors like Yin Zhang et al. and Qian Zhou et al. respectively, this observation was consistent with our results [4, 5]. Preventive strategies for pediatric surgery of meatal stenosis include UP incision, not extending too distally, and not suturing the neourethra to the glanular wings. Both of these preventive measures were adopted in our techniques. The limitations of this study are as follows: First, despite the high level of experience possessed by both operators in hypospadias surgery, the intricacies of their surgical techniques and potential measurement bias could still impact the study’s outcome. Second, patients had short- to medium-term follow-up results provided, and there is no literature reporting the long-term efficacy of Buck’s fascia repair. Therefore, further follow-up must be conducted in the future to supplement the long-term efficacy of Buck’s fascia repair.

## Conclusion

Our results indicate that the utilization of Buck’s fascia as an intermediate barrier between the neourethra and the skin offers several advantages. Buck’s fascia repair was found to be significantly less time-consuming due to minimal dissection, resulting in a reduction in early post-operative problems such as glanular edema, hematoma, and wound infection, as compared to the dartos flap. Our findings suggest that Buck’s fascia offers a promising alternative to traditional interposition layers and holds the potential to improve surgical outcomes in hypospadias repair, addressing the primary goal of reducing fistula formation and meatal stenosis. Further research and longer-term follow-up studies may help confirm and expand upon these promising results.

## Data Availability

Data is provided within the manuscript.
